# Validity of Diagnostic Codes and Prevalence of Physician-Diagnosed Psoriasis and Psoriatic Arthritis in Southern Sweden – A Population-Based Register Study

**DOI:** 10.1371/journal.pone.0098024

**Published:** 2014-05-29

**Authors:** Sofia Löfvendahl, Elke Theander, Åke Svensson, Katarina Steen Carlsson, Martin Englund, Ingemar F. Petersson

**Affiliations:** 1 Department of Orthopedics, Clinical Sciences Lund, Lund University, Lund, Sweden; 2 Epidemiology and Register Centre South, Skåne University Hospital, Lund, Sweden; 3 Department of Rheumatology, Skåne University Hospital Malmö, Lund University, Malmö, Sweden; 4 Department of Dermatology, Skåne University Hospital Malmö, Lund University, Malmö, Sweden; 5 Department of Clinical Sciences, Malmö, Lund University, Skåne University Hospital Malmö, Sweden; 6 Clinical Epidemiology Research & Training Unit, Boston University School of Medicine, Boston, Massachusetts, United States of America; 7 Arthritis Research UK Primary Care Centre, Keele University, United Kingdom; University of Birmingham, United Kingdom

## Abstract

**Objective:**

To validate diagnostic codes for psoriasis and psoriatic arthritis (PsA) and estimate physician-diagnosed prevalence of psoriasis and PsA in the Skåne region, Sweden.

**Methods:**

In the Skåne Healthcare Register (SHR), all healthcare consultations are continuously collected for all inhabitants in the Skåne region (population 1.2 million). During 2005–2010 we identified individuals with ≥1 physician-consultations consistent with psoriasis (ICD-10). Within this group we also identified those diagnosed with PsA. We performed a validation by reviewing medical records in 100 randomly selected cases for psoriasis and psoriasis with PsA, respectively. Further, we estimated the pre- and post-validation point prevalence by December 31, 2010.

**Results:**

We identified 16 171 individuals (psoriasis alone: n = 13 185, psoriasis with PsA n = 2 986). The proportion of ICD-10 codes that could be confirmed by review of medical records was 81% for psoriasis and 63% for psoriasis with PsA with highest percentage of confirmed codes for cases diagnosed ≥2 occasions in specialized care. For 19% and 29% of the cases respectively it was not possible to determine diagnosis due to insufficient information. Thus, the positive predicted value (PPV) of one ICD-10 code for psoriasis and psoriasis with PsA ranged between 81–100% and 63–92%, respectively. Assuming the most conservative PPV, the post-validation prevalence was 1.23% (95% CI: 1.21–1.25) for psoriasis (with or without PsA), 1.02% (95% CI: 1.00–1.03) for psoriasis alone and 0.21% (95% CI: 0.20–0.22) for psoriasis with PsA. The post-validation prevalence of PsA in the psoriasis cohort was 17.3% (95% CI: 16.65–17.96).

**Conclusions:**

The proportion of diagnostic codes in SHR that could be verified varied with frequency of diagnostic codes and level of care highlighting the importance of sensitivity analyses using different case ascertainment criteria. The prevalence of physician-diagnosed psoriasis and PsA confirm other population-based studies, also after adjustment due to misclassification of disease.

## Introduction

Psoriasis is a chronic, inflammatory disease mainly affecting the skin and nails. There are different types of psoriasis, but the most common form is plaque psoriasis. Family history as well as environmental factors such as infections, mental stress, alcohol and smoking, skin injuries and certain medications can trigger the disease [1], [2]. A number of those with psoriasis problems also develop psoriatic arthritis (PsA). PsA is thus also a chronic inflammatory disease with major manifestations as inflammatory arthritis, enthesitis, tenosynovitis, and spondyloarthritis resulting in pain, stiffness and swelling in and around the joints or in the back. Most people (60–75%) who develop PsA already have a diagnosed psoriasis. In 10–15% of the cases, inflammatory arthritis is the first symptom and simultaneously onset of arthritis and skin disease occurs with approximately the same frequency [3], [4]. As these diseases are chronic and often affect individuals of working age there are implications not only for the individuals, but also for society in terms of health care costs and costs due to productivity losses [5]–[10].

Studies from across the world have reported prevalence estimates of psoriasis and PsA ranging from 0.7% to 3.2% and 0.1% to 0.42% respectively during the last decade [11], [12]. The proportion of psoriasis patients suffering from PsA has varied between 7% and 31% [13]–[15]. These differences may be explained by differences in methodology (differences in case definition, target population, sample size, age or ethnicity) but also by insufficient or incorrect validation procedures.

Accurate and timely population-based prevalence estimates are of importance in the understanding of the psoriasis and psoriatic arthritis burden of disease and time trends [8], [16]. Many of the existing prevalence studies are of small sample sizes from clinic cohorts or disease registers without appropriate coverage of a whole population, but studies using population-based administrative or medical record databases are increasing in number [16]–[18]. One large benefit of using register-based information is also the rapid access to large amounts of data at low cost, which make it possible to perform parallel studies using a single data source. However, this should include strict and proper validation procedures for case ascertainment which is most often not the case.

One commonly used method for case ascertainment in the research on specific disease groups using healthcare registers is to rely on medical diagnostic codes [8], [16], [17]. An important issue to address in this context is the validity of the diagnostic codes used to identify the patients in question. So far, there are only few studies reporting on the validity of diagnostic codes for psoriasis and PsA in population-based healthcare registers [16]–[20].

The population-based Skåne Healthcare register (SHR) in Sweden has been used in various studies addressing the validity of diagnostic codes and burden of disease perspective as the register offers a great opportunity in the longitudinal coverage on all health care utilization (both primary care and specialized care), registered with diagnostic codes according to the Swedish version of the International Classification of Diseases and Related Health Problems system, version 10 (ICD-10-SE), for a large and well-characterized population [21]–[24].

The aim of this study was to investigate the validity of the ICD-10-SE diagnostic codes for psoriasis and PsA in the SHR. Our hypothesis was that the level of correspondence between the diagnostic code in the SHR and the information retrieved from the medical records may vary depending on level of care (primary care versus specialized care) and how frequent the code appeared within the same patient. We also estimated the prevalence of physician-diagnosed psoriasis and PsA, pre- and post-validation, using the SHR.

## Methods

### Study Setting

In this study the target population included all residents in the Skåne region in southern Sweden and the data sources used were the SHR and the Swedish population register.

#### Ethics statement

This study was conducted according to the Declaration of Helsinki and approved by the Regional Ethical Review Board in Lund, Sweden (Dnr 301/2007, Dnr 406/2008 and supplement to Dnr 2012/359). According to the Ethical Review Board decision and in line with Swedish law, all individuals eligible for inclusion in the study were informed in the regional news press and offered an opt-out opportunity. After the linkage, all data were analysed anonymously. For the review of the medical records, consent was obtained from the medical director/physician responsible for each patient. The data extracted from the medical records were anonymized prior to analysis.

#### Target population and health care system

The Skåne region is located in the southernmost part of Sweden and holds both rural and urban areas. Its population, 1 242 079 inhabitants by 31 November 2010, accounts for approximately one-eighth of the Swedish population [21]. The Swedish health care is predominantly tax-financed with user fees and private insurances covering 16% and 0.26% of the total health care expenditures respectively [25]. The SHR is used as the basis for reimbursement of both public and private health care providers.

#### SHR

SHR contains information transferred from both computerized medical records and from administrative application sources on all health care utilization in the Skåne region. In the register, data on all primary care and specialized outpatient and inpatient care is continuously collected for individuals living in the Skåne region including personal identification number (PIN), age, sex, health care provider (physician, nurse, physiotherapist and other), date of visit and diagnostic codes according to ICD-10-SE. Private and public care are registered exactly in the same way in SHR except for the diagnostic codes in private care which are not forwarded to the SHR.

#### Population register

The Swedish national population register is the civil registration of vital events (e.g. births, deaths, marriages, residential area) of all Swedish inhabitants. The register is continuously updated and used for a variety of purposes by official authorities and by health care providers. In the register, all citizens are identified by their unique 10-digit PIN. By law, all in specialized in- and outpatient care (primary health care excluded) provided has to be registered by the individual’s PIN. Through this it is possible to link information from the Swedish population register to SHR and vice versa.

### Inclusion Criteria

From the SHR we selected individuals who, at any time during the period 1 January 2005 to 31 December 2010, had consulted any physician and been given an ICD-10 diagnostic code indicating psoriasis. From this group we thereafter selected those who also had a physician consultation where they had received a diagnostic code indicating PsA.

To define the case criteria for psoriasis and PsA we used the ICD-10-SE diagnostic codes registered in the SHR. For each physician consultation we used the first eight of up to 15 possible diagnostic codes positions to search for relevant cases.

Diagnostic codes considered for psoriasis were L40.0, L40.1, L40.2 L40.4, L40.5, L40.8 and L40.9 (see Table 1 for text explanation of the codes). We required each psoriasis case to have consulted a physician at least once in between 1 January 2005 and 31 December 2010 and having been diagnosed with any of the ICD-10 diagnosis codes consistent with psoriasis (see above).

**Table 1:** ICD-10 codes used to identify cases of psoriasis and psoriatic arthritis in the Skåne Healthcare Register.

ICD-10 code	Diagnosis full text
L40.0	Psoriasis vulgaris
L40.1	Generalized pustular psoriasis
L40.2	Acrodermatitis continua
L40.4	Guttate psoriasis
L40.5	Arthropathic psoriasis
L40.8	Other psoriasis
L40.9	Psoriasis unspecified
M07.0	Distal interphalangeal psoriatic arthropathy
M07.1	Arthritis mutilans
M07.2	Psoriatic spondylitis
M07.3	Psoriatic arthropaties
M09.0	Juvenile arthritis in psoriasis

Individuals diagnosed with “arthropathic psoriasis” (ICD-10 diagnostic code L40.5) were directly qualified as PsA cases. Among the rest of the psoriasis cases the prerequisite to qualify as a PsA case was further registration of at least one of the ICD-10 diagnostic codes M07.0, M07.1, M07.2, M07.3 or M09.0 (see Table 1 for text explanation of the codes). Individuals of all ages were included.

### Validation of Diagnostic Codes for Psoriasis and PsA

**Figure 1:**
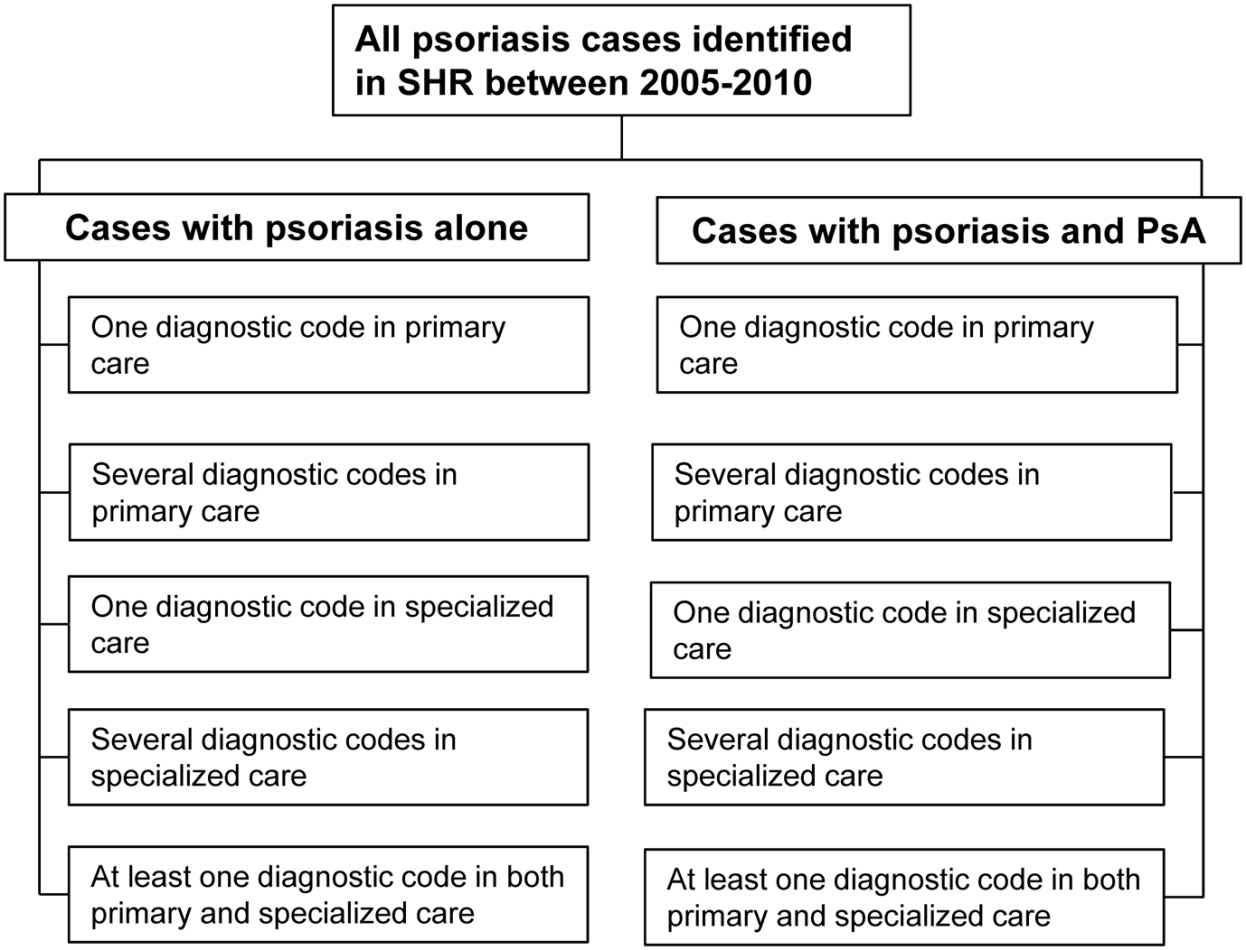
Psoriasis and PsA cases identified in the Skåne Healthcare Register (SHR) during the period 2005 to 2010 and groups of cases with psoriasis alone and cases with psoriasis and PsA. Each group subdivided according to the frequency of diagnostic codes and level of care.

The validation of ICD 10 diagnostic codes was performed for two groups of patients (Figure 1): those with psoriasis alone and those with psoriasis and PsA. In the latter group we validated only the diagnostic codes consistent with PsA.

The cases with psoriasis alone and psoriasis with PsA were divided into five subgroups respectively according to level of care and how frequent the code appeared within the same patient (frequency of diagnostic codes) during the six year study time window (2005–2010). The five subgroups were: 1) primary care -1 diagnostic code only, 2) primary care - ≥2 diagnostic codes, 3) specialized care -1 diagnostic code only, 4) specialized care - ≥2 diagnostic codes and 5) at least one code in both primary and specialized care (Figure 1). In each subgroup, 20 cases were selected at random, which in total added up to 100 selected cases for the validation of the diagnostic codes for psoriasis alone and psoriasis with PsA, respectively.

For all physician visits (any physician) registered in SHR at which the patients had received any diagnostic code consistent with psoriasis or PsA during the period 2005–2010, the corresponding medical record notes were thoroughly read for validation of whether the diagnostic code captured in the SHR truly reflected psoriasis and PsA.

Information from the primary care medical records was delivered on paper while information from the specialized care medical record was made available electronically. For the cases with a diagnostic code for psoriasis or PsA both in primary care and specialized care we started by reviewing the specialized care medical records. For the review of the medical records we used two separate extraction forms, one for the psoriasis cases and one for the PsA-cases. Both forms were developed by the authors of whom one is an experienced dermatologist (ÅS) and two are experienced rheumatologists (ET and IP). The form used for the psoriasis cases consisted of questions regarding heredity, rash, scaling, nail involvement and localization of skin changes. In addition to this it was possible to include other information, e.g. patient history and pharmaceutical treatment from the medical record with relevance for the verification of the psoriasis diagnosis. Based on this information it was decided whether the psoriasis diagnosis was 1) verified, 2) unverified due to insufficient information or 3) verified as a non-psoriasis diagnosis. In the form used for the validation of the diagnostic codes for the PsA-cases the classification Criteria for Psoriatic Arthritis (CASPAR) were used as the standard [26]. For cases not fulfilling the 3 points needed in the CASPAR chart to qualify as a PsA it was still possible to qualify as a PsA-case if the medical records included additional information with relevance for the verification of the PsA diagnosis. Based on the information in the predefined form it was decided whether the PsA diagnosis was 1) verified from an overall assessment of the medical record, 2) not verified due to insufficient information in the medical record, 3) verified as a non-PsA diagnosis. Finally, we applied the CASPAR criteria alone. The reviews of the medical records and a preliminary completion of the extraction forms were performed by an external physician with experience form both the dermatology and rheumatology field. After this initial phase, one dermatologist (ÅS) and one rheumatologist (ET) reviewed all the forms for psoriasis and PsA and made the final decision regarding the accuracy of the diagnosis. In cases of ambiguity, the specialized physicians reviewed the medical records again.

### Prevalence Estimates of Physician-diagnosed Psoriasis and PsA

Using data from the SHR, the inclusion criteria were applied to identify all individuals diagnosed with psoriasis and PsA at any time during the period from 1 January 2005 to 31 December 2010. By means of the individuals’ PIN, data were linked from the SHR to the Swedish population register to exclude those who were no longer alive or no longer residents in the county by the end of 2010. Hence, the point prevalence of physician-diagnosed psoriasis and PsA was estimated by dividing the number of individuals who met our inclusion criteria by the number of residents living in the Skåne region by 31 December 2010. We presented the prevalence of 1) psoriasis with or without PsA, 2) psoriasis alone and 3) psoriasis with PsA for the Skåne region population. In addition, the prevalence of PsA-cases with psoriasis in the population of psoriasis cases was also presented. In the calculation of the prevalence estimates, the figure used for the number of residents living in Skåne was reduced by 15% to adjust for the uncertainty generated by the loss of patients consulting *only* private practitioners and whose diagnoses are not forwarded to the register (although the patients PIN and date of consultations are). Hence, the denominator used was a population of 1 055 766 inhabitants. For a detailed reasoning behind the magnitude of the deduction used, see a previous article from our group [22].

### Statistical Analyses

First, we presented descriptive data on demographic variables, patterns of diagnostic codes and physician-consultations for the individuals included in the study. Second, we validated the diagnostic codes of psoriasis and PsA reported in the SHR. When the information in the medical record supported the ICD-10-SE diagnostic code registered in the SHR, the diagnostic code was assumed to be confirmed. We presented the proportion of correct diagnostic codes (the positive predicted value - PPV) in the SHR out of the cases selected for validation and for which it was possible to obtain the medical records.

Finally, pre- and post-validation prevalence estimates of psoriasis and PsA were calculated. The post-validation estimates were based on the most conservative estimate of the positive predictive values of the diagnostic codes for psoriasis and PsA. We also assumed no misclassification of psoriasis and PsA in the other direction in SHR. 95% confidence intervals around the prevalence estimates were calculated using a binomial distribution. The analyses were performed using Stata Statistical Software (Release 10. College station, TX: StataCorp LP) and SPSS.

## Results

### Population Characteristics

During the six-year study period (2005–2010), we identified 16 171 individuals who fulfilled the inclusion criteria, i.e., diagnosed with psoriasis and/or PsA. Out of those, 13 185 had at least one diagnostic code consistent with psoriasis and 2 986 had at least one diagnostic code consistent also with PsA (Table 2). Of the cases with psoriasis alone 6 488 (49.2%) were women. The corresponding figure for cases with psoriasis and PsA was 1 706 (57.1%). For both sexes, the mean age as on December 31 2010 was slightly lower for cases with psoriasis alone compared to those with psoriasis and PsA.

**Table 2:** Characteristics of the cases identified and diagnostic code pattern during 2005–2010.

	Cases with psoriasis alone (N = 13 185)	Cases with psoriasis and PsA (N = 2 986)	All cases (N = 16 171)
**Women (%)**	6488 (49.2)	1706 (57.1)	8194 (50.7)
**Age, mean (SD)**			
Women	53 (21)	55 (16)	53 (19.8)
Men	53 (19)	55 (15)	53 (18.2)
**Annual number of doctor-consultations consistent with psoriasis and PsA during 2005–2010, mean (SD)**			
All	0.47 (0.58)	1.57 (1.73)	0.68 (1.0)
Women	0.47 (0.54)	1.62 (1.84)	0.71 (0.65)
Men	0.49 (0.61)	1.51 (1.58)	0.65 (0.93)
**Number of cases with different diagnostic codes related to psoriasis (%)**			
L40.0	4 565 (34.6)	800 (26.8)	5 365 (33.2)
L40.1	52 (0.4)	14 (0.5)	66 (0.4)
L40.2	45 (0.3)	2 (0.1)	47 (0.3)
L40.3	377 (2.9)	183 (6.1)	560 (3.5)
L40.4	419 (3.2)	33 (1.4)	452 (2.8)
L40.5	–	2 948 (98.7)	2 948 (18.2)
L40.8	233 (1.8)	57 (1.9)	290 (1.8)
L40.9	10 548 (80.0)	1 322 (44.3)	11 870 (73.4)
**Number of cases with different diagnostic codes related to** **“psoriatic and enteropathic arthropathies” or** **“juvenile arthritis in psoriasis” (%)**			
M07.0	–	13 (0.4)	13 (0.4)
M07.1	–	153 (5.1)	153 (5.1)
M07.2	–	61 (2.0)	61 (2.0)
M07.3	–	1 223 (41.0)	1 223 (41.0)
M09.0	–	102 (3.4)	102 (3.4)
**Number of cases with a primary diagnostic code for psoriasis or PsA**	10 005 (75.9)	2 719 (91.8)	12 721 (78.1)
**Number of cases with a dermatologist, rheumatologist or internist consultation (%)**	7 703 (58.4)	2 634 (88.2)	10 337 (63.9)

#### Psoriasis alone

Of the 13 185 cases with psoriasis alone, the most commonly used diagnostic code for psoriasis was L40.9 “psoriasis, unspecified” in 10 548 (80.0%) of the cases followed by L40.1 “Psoriasis vulgaris” in 4 565 (34.6%) (Table 2). During the study period there were 37 888 physician consultations consistent with a psoriasis diagnostic code registered in the SHR for the cases with psoriasis alone. This corresponds to a mean (SD) of 0.47 (0.58) doctor-consultations per year and case (Table 2). A diagnostic code for psoriasis as primary code were registered for 10 005 (75.9%) of the cases and 7 703 (58.4%) had at least one psoriasis diagnostic code given by a dermatologist, rheumatologist or internist.

#### Psoriasis with PsA

Of the 2 986 cases with both psoriasis and PsA the most commonly used diagnostic code for psoriasis and also for PsA (by definition in this study) was L40.5 “Arthropathic psoriasis” in 2 948 (98.7%) of the cases. The cases, 38 (1.3%), who did not have a diagnostic code of L40.5 registered qualified as PsA cases having another diagnostic code for psoriasis in combination with any “M07-code” (data not shown). The most common “M07” diagnostic code was M07.3 “Psoriatic arthropathies” in 1 223 (41%) of the cases (Table 2). There were 28 143 physician consultations consistent with psoriasis and PsA registered in the SHR for this group of cases. This means a mean (SD) of 1.57 (1.73) physician-consultations per year and case (Table 2). A diagnostic code for psoriasis and PsA as primary code were registered for 2 719 (91.8%) of the cases and 2 634 (88.2%) had at least one psoriasis and PsA diagnostic code given by a dermatologist, rheumatologist or internist.

### Validation of the of ICD-10 Diagnostic Codes for Psoriasis and PsA in the SHR

#### Psoriasis alone

Out of the 13 185 cases identified as cases with psoriasis alone during 2005–2010, 3 349 (25.5%) had received a diagnostic code for psoriasis on a single occasion in primary care and 1 481 (11.2%) on several occasions in primary care. The majority of the cases, 8 355 (63.4%) had received a psoriasis diagnostic code at least once in specialized care (Table 3).

**Table 3:** Level of care and frequency of diagnostic codes for the cases identified as psoriasis alone in Skåne Healthcare Register 2005–2010; validation of diagnostic codes by review of medical records for a subsample of these cases.

Review of medical records for diagnostic code validation of psoriasis cases					
Level of care and frequencyof psoriasisdiagnostic codes†	Pre-validation number of caseswith psoriasisalone (%) inthe Skåneregionpopulation	Review of medical record			Adjusted†† numbers of cases withpsoriasis alone (%) in the Skåne regionpopulation based onvalidation
		Number of casesactually reviewed	Dermatologist confirmedpsoriasis cases, n (%)	Insufficient information fordiagnosis verification, n (%)	
One code in primary care only	3 349 (25.5)	23*°	15/23 (65)	8/23 (35)	2 184 (20.4)
Two or more codes in primary care only	1 481 (11.2)	14*°	11/14 (79)	3/14 (21)	1 164 (10.9)
One code in specialized care only	2 828 (21.4)	20	14/20 (70)	6/20 (30)	1 980 (18.5)
Two or more codes in specialized care only	2 771 (21.0)	20	20/20 (100)	0/20 (0)	2 771 (25.9)
One or more codes in *both* primary and specialized care	2 756 (20.9)	20	19/20 (95)	1/20 (5)	2 618 (24.4)
All cases	13 185 (100)	97°	79/97 (81)	18/97 (19)	10 717 (100)

† Any code consistent with psoriasis.

†† Adjustment based on the proportion of correct diagnostic codes (the positive predicted value) of psoriasis in the SHR.

*4 cases found to be misclassified (moved from ≥2 primary care codes to 1 primary care code) °Medical records for 3 cases (one primary care 1 code and two primary care ≥2 codes) were impossible obtain due to administrative reasons.

Out of the cases with psoriasis alone, 100 (0.76%) cases were selected for validation according to level of care and frequency of diagnostic codes (Table 3). For three cases, the medical notes were missing and validation could not be performed. Overall, at least 79 of 97 (81%) of the validated psoriasis cases were registered with a correct diagnostic code in SHR. For the rest of the 18 cases (19%), description of lesions and patient history were not sufficient for an assessment whether it was psoriasis or not. Thus, the PPV of an ICD-10-SE psoriasis diagnostic code was within the range of 81% to 100%. The number of dermatologist confirmed psoriasis cases increased in the presence of more than one diagnostic code in both primary and secondary care (Table 3).

#### Psoriasis with PsA

Out of the cases with psoriasis and PsA, 137 (4.6%) cases had received a diagnostic code for PsA on a single occasion and 114 (3.8%) cases on several occasions in primary care (Table 4). The majority of the cases, 2 735 (91.6%), had received a PsA diagnostic code at least once in specialized care.

**Table 4:** Level of care and frequency of diagnostic codes for the cases identified as psoriasis with psoriatic arthritis (PsA) in Skåne Healthcare Register 2005–2010; validation of diagnostic codes by review of medical records for a subsample of these cases.

Review of medical records for diagnostic code validation of cases with psoriasis and PsA							
Level of care and frequencyof PsA diagnostic codes†	Pre-validation number of caseswith psoriasis and PsA (%)in the Skåne regionpopulation	Review of medical record					Adjusted†† numbers of caseswith psoriasis and PsA (%)in the Skåne region populationbased on validation
		Number of casesactually reviewed	Rheumatologist confirmed(overall assessment of medical record)PsA cases, n (%)	Insufficient information fordiagnosis verification, n (%)	Verified asa non-PsAdiagnosis,n (%)	Confirmed cases when CASPARclassification criteria was applied solely,n (%)	
One code in primary care only	137 (4.6)	17°	9/17 (53)	6/17 (35)	2/17 (12)	4/17 (24)	72 (3.2)
Two or more codes in primary care only	114 (3.9)	17°	9/17 (53)	7/17 (41)	1/17 (6)	3/17 (18)	60 (2.7)
One code in specialized care only	658 (22.0)	20	10/20 (50)	8/20 (40)	2/20 (10)	4/20 (20)	329 (14.7)
Two or more codes in specialized care only	1 670 (55.9)	19°	17/19 (89)	1/19 (5)	1/19 (5)	14/20 (74)	1 495 (66.7)
One or more codes in *both* primary and specialized care	407 (13.6)	20	14/20 (70)	5/20 (25)	1/20 (5)	11/20 (55)	285 (12.7)
All cases	2 986 (100)	93°	59/93 (63)	27/93 (29)	7/93 (8)	36/20 (39)	2 241 (100)

† Any code consistent with PsA.

†† Adjustment based on the proportion of correct diagnostic codes (the positive predicted value) of PsA in the SHR °Medical records for 7 cases (three primary care 1 code, three primary care ≥2 codes and one specialized care ≥2 codes) were impossible obtain due to administrative reasons.

In the group of cases with psoriasis and PsA, 100 (3.3%) were selected for the validation (Table 4). Of these seven could not be retrieved due to administrative reasons. The minimal number of correctly recorded cases with psoriasis and PsA according to the overall assessment of the medical records was found to be 59 of 93 (63%). For an additional 27 cases (29%), the information in the medical record was not sufficient to ascertain whether it was psoriasis with PsA or not. Thus, the positive predicted value of an ICD-10 PsA diagnostic code was within the range of 63% to 92%. Seven cases (8%) had probably another diagnosis, e.g. rheumatoid arthritis, gout or osteoarthritis with psoriasis. The number of cases that strictly fulfilled the CASPAR classification criteria (solely based on information in medical records) was 36 (39%). The proportion of confirmed cases increased with at least one code in both primary care and specialized (Table 4). The increase was even more accentuated for cases with several diagnostic codes rendered in specialized care.

### Doctor-diagnosed Prevalence of Psoriasis and PsA

#### Prevalence estimates pre-validation

Table 5 presents the pre-validation prevalence estimates of physician-diagnosed psoriasis and PsA for individuals of all ages in the Skåne region population by the end of 2010. The overall prevalence of psoriasis (with or without PsA) cases was 1.53% (95% confidence interval [CI]: 1.51–1.56). The corresponding figure for cases with psoriasis alone was 1.25% (95% CI: 1.23–1.27). The overall prevalence of cases with psoriasis and PsA was 0.28% (95% CI: 0.27–0.29). The prevalence of PsA cases among individuals with psoriasis was 18.5% (95% CI: 17.81–19.14). In the group of cases with psoriasis alone the prevalence was slightly higher for men compared with women. The opposite relation was true for cases with psoriasis and PsA.

**Table 5:** Pre-validation prevalence estimates of doctor-diagnosed psoriasis and psoriatic arthritis (PsA) by sex in the Skåne region by December 31, 2010.

	Prevalence % of psoriasis and PsA in the Skåne region population (95% CI)			Prevalence of PsA in the psoriasis cohort
	All psoriasis cases (n = 16 171)in the Skåne region pop.(N = 1 055 766)	Psoriasis alone (n = 13 185)in the Skåne region pop.(N = 1 055 766)	Psoriasis with PsA (n = 2 986)in the Skåne region pop.(N = 1 055 766)	Psoriasis with PsA (n = 2 986)in the psoriasis cohort(N = 16 171)
Women	1.54 (1.50–1.57)	1.22 (1.19–1.25)	0.32 (0.31–0.34)	20.8 (19.95–21.72)
Men	1.53 (1.49–1.56)	1.28 (1.25–1.31)	0.24 (0.23–0.26)	16.0 (15.25–16.87)
All cases	1.53 (1.51–1.56)	1.25 (1.23–1.27)	0.28 (0.27–0.29)	18.5 (17.87–19.07)

#### Adjusted prevalence estimates

Using the most conservative estimate of the positive predicted value, the post-validation overall prevalence of psoriasis and psoriasis with PsA was 1.23% (95% CI: 1.21–1.25). The corresponding figure for cases with psoriasis alone was 1.02% (95% CI: 1.00–1.03). The adjusted prevalence figure for cases with psoriasis and PsA was 0.21% (95% CI: 0.20–0.22). The prevalence of PsA cases in the psoriasis population was adjusted slightly downwards to 17.3% (95% CI: 16.65–17.76) (Table 6). The validation did not change the relative magnitude of the prevalence estimates across sexes.

**Table 6:** Post-validation prevalence estimates of doctor-diagnosed psoriasis and PsA in the Skåne region by December 31, 2010 based on a conservative positive predictive value.

	Prevalence % of psoriasis and PsA in the Skåne region population (95% CI)			Prevalence of PsA in the psoriasis cohort
	All psoriasis cases (n = 12 958)in the Skåne region pop.(N = 1 055 766)	Psoriasis alone (n = 10 717)in the Skåne region pop.(N = 1 055 766)	Psoriasis with PsA (n = 2 241)in the Skåne region pop.(N = 1 055 766)	Psoriasis with PsA (n = 2 241)in the psoriasis cohort(N = 12 958)
Women	1.23 (1.20–1.26)	0.99 (0.96–1.02)	0.24 (0.23–0.25)	19.5 (18.54–20.47)
Men	1.22 (1.19–1.25)	1.04 (1.01–1.07)	0.18 (0.17–0-20)	15.0 (14.17–15.93)
All cases	1.23 (1.21–1.25)	1.02 (1.00–1.03)	0.21 (0.20–0.22)	17.3 (16.65–17.96)

## Discussion

In this study, we performed a validation of the ICD-10-SE diagnostic codes registered for psoriasis and PsA in the population-based SHR versus the medical records, and the results showed that the proportion of diagnostic codes that could be verified was at least 81% and 63% respectively. Due to lack of information in the medical records it was not possible to assess whether the diagnostic code was correct or not in 19% and 29% of the reviewed cases of psoriasis and PsA, respectively. Thus, the PPV of an ICD-10-SE psoriasis and psoriasis with PsA diagnostic code was within the range of 81% to 100% and 63% to 92%, respectively.

As we hypothesized, the number of confirmed diagnoses varied with level of care and how frequent the code appeared within the same patient, with the highest percentage of confirmed diagnostic codes for cases evaluated on several occasions in specialized care. We also estimated the prevalence of psoriasis and PsA, both unadjusted and, adjusted for potential false positives in the SHR, i.e. misclassified as to have psoriasis and/or PsA. The pre-validation (unadjusted figures) physician-diagnosed prevalence was 1.53% (95% CI: 1.51–1.56) for psoriasis with or without PsA, 1.25% (95% CI: 1.23–1.27) for psoriasis alone and 0.28% (95% CI: 0.27–0.29) for psoriasis with PsA in individuals of all ages in the approximately 1.2 million large population of the Skåne region. The prevalence of PsA in the psoriasis population was estimated to 18.5% (95% CI: 17.8–19.1). After adjustment of the estimates, assuming the most conservative PPV due to misclassification of diagnostic codes, the prevalence figures still remained in line with results from other population-based psoriasis and PsA prevalence studies.

A main critic towards population-based studies using administrative database sources is the trust on different types of diagnostic codes for case ascertainment as they may not reflect the patients’ true conditions [8]. Our results suggested correctly recorded diagnostic codes in at least 81% of the psoriasis cases with the true PPV probably higher. The minimum proportion of correctly recorded diagnostic was lower for those with psoriasis diagnostic code on a single occasion (67%) and higher for those with a diagnostic code on several occasions (93%). These results are supported by an evaluation of psoriasis diagnostic codes indexed in an US electronic database in the setting of Olmsted County Minnesota where the correctness of a single code for psoriasis was 60% with an increase in the correctness for more than one code over time [17]. Similarly high accuracy of diagnostic codes for psoriasis as in SHR has been shown in an electronic primary care database (The Health Improvement Network -THIN) in the UK (90%) [18] and in a managed care organization setting in northern California in the US (89%) [16].

A common case ascertainment method for psoriasis cases is to rely on dermatologist confirmed diagnosis which means that mostly cases given the diagnosis in specialized care are included. Our study showed that there is a risk of excluding true psoriasis cases from a study population relying on this method as many patients actually consult only primary care and also get a correct diagnosis there. We showed that in the Skåne region nearly one third of those with psoriasis consulted only primary care physicians and received a correct diagnosis. An American study based on self-reported data described that 78.3% of the psoriasis patients consulted a specialist and 22% received care from primary care physicians [27].

The minimum number of correctly recorded cases with psoriasis and PsA was found to be 59 (63%). Limited information in the medical records was a problem and in an additional 29% we could neither confirm nor disconfirm the diagnosis. The proportion of correctly recorded cases increased to 89% for cases with a diagnostic code for psoriasis and PsA on at least two occasions in specialized care. This result is supported by the US study mentioned above, where the investigators also validated the diagnostic codes for cases with psoriasis and PsA reporting a proportion of correct diagnosis for 64% for PsA cases with at least one diagnostic code for PsA rendered by any physician [16]. In the US study, the proportion of diagnoses that could be confirmed increased to 71% for the cases with at least one diagnostic code given by both a dermatologist and rheumatologist. In the THIN cohort, out of 100 patients seeing a general practitioner with a Read Code for PsA, 74 (85%) was reported to be correctly diagnosed. The majority, 62 (84%), of these patients had also been seen by a rheumatologist [14].

In our material, more diagnoses that could be verified to be correct were observed for those who received a diagnostic code consistent with PsA at least once in both primary care and specialized care (70%) or on several occasions in specialized care (89%). At the same time, only 45% of the cases with a diagnostic code for PsA in primary care could be verified. These results suggest that PsA can be difficult to diagnose; it is a heterogeneous disease and symptoms may be similar to those seen in other rheumatic diseases [14], [28], and indicates that PsA seems to be a disease that needs to be confirmed at least two times, including one time in specialized care.

To our knowledge, this is the first Swedish study in recent years addressing population-based estimates of physician-diagnosed psoriasis with or without physician-diagnosed PsA with a proper validation process against medical records. A Swedish study from 1967, using clinical examination as case ascertainment method, reported a psoriasis prevalence of 1.9% [29]. Worth noticing is also a large UK study by Gelfand and co-workers which estimated the prevalence of psoriasis for individuals of all ages to 1.5% in a general population of nearly 7.5 million people [30]; a result similar to that of our study. The above results are also confirmed by other studies taking a population-based perspective. In a systematic review by Parisi et al. the psoriasis prevalence for individuals of all ages in different European populations ranged from 0.7% to 2.9%, with most prevalence results around 2%. Rates from the US range from 0.7% to 2.6% [12].

Regarding the prevalence of PsA, there are fewer population-based studies compared to psoriasis. Our estimated prevalence of 0.28% (unadjusted) and 0.21% (adjusted) is supported by Gelfand and colleagues estimating the prevalence of PsA to 0.25% in a sample of the US general population [31]. However, in contradiction to our study, they used a relatively small adult target population which brings a certain amount of uncertainty into the prevalence estimates. Lower prevalence estimates have been reported in population-based studies from Norway (0.13%) [32], Denmark (0.15%) [33] and the US (0.1%) [34], [35]. Another Swedish study, also using the SHR, estimating the prevalence of PsA in the population of the Skåne region, reported a prevalence of 0.25% which is analogous to our result. This study took a starting point in the spondyloarthritis disease group were PsA is one of the subtype conditions. Hence the inclusion criteria and time period differed somewhat compared with the present study where the psoriasis population was the basis [22].

Our prevalence estimate of 18.5% (unadjusted) and 17.3% (adjusted) PsA cases among patients with psoriasis is the mid-range compared to other western studies of recent date. A clinic-based German study and a population-based UK study suggested prevalence figures of 19% and 14% respectively [36], [37]. Higher prevalence estimates of PsA in psoriasis patients have been reported in both patient organization-based and clinic-based studies [13], [38], [39].

The attractiveness of this study compared with many other studies on the prevalence of psoriasis and PsA is that we have been able to estimate the prevalence using a single data source covering both primary care and specialized care utilization for a large population. Limitations of the study include the fact that there may be an underestimation of the true prevalence of psoriasis and PsA in the Skåne due to several reasons. First, only individuals only consulting a healthcare provider (physician) for their psoriasis or PsA problems were included. Second, we did not evaluate whether there were cases misclassified as not to have psoriasis and/or PsA (false negatives) in the SHR. Third, we did assume the most conservative positive predicted value of an ICD-10-SE code for psoriasis and psoriasis with PsA. Fourth, we did not include individuals with PsA but without psoriasis. However, one circumstances that may have contributed to a slightly overestimation of the true prevalence is that the reviewing physicians, a priori the reading of the medical records, knew about the psoriasis and PsA diagnosis of the individuals. This may have led to a higher share of confirmed diagnosis compared to if the physicians would have been unaware of the diagnosis.

In conclusion, results support the SHR to be a valid healthcare register for studies on psoriasis and PsA. However, the proportion of the diagnostic codes that could be verified varied with frequency of diagnostic codes and level of care, which highlight the usefulness to perform sensitivity analyses using different criteria for case ascertainment. Furthermore, we have received a robust measure of the impact of psoriasis and PsA in terms of physician-diagnosed prevalence using a validation against medical records. Assuming a number of conservative scenarios, also the post-validation prevalence estimates of psoriasis and PsA can confirm results from other population-based studies.

## References

[1] Richardson SK, Gelfand JM (2008) Update on the natural history and systemic treatment of psoriasis. Adv Dermatol 24: 171–196. 19256309 10.1016/j.yadr.2008.09.006 PMC2634854

[2] Swanbeck G, Inerot A, Martinsson T, Wahlstrom J (1994) A population genetic study of psoriasis. Br J Dermatol 131: 32–39. 8043420 10.1111/j.1365-2133.1994.tb08454.x

[3] Biondi Oriente C, Scarpa R, Pucino A, Oriente P (1989) Psoriasis and psoriatic arthritis. Dermatological and rheumatological co-operative clinical report. Acta Derm Venereol Suppl (Stockh) 146: 69–71. 2609889

[4] Scarpa R, Oriente P, Pucino A, Torella M, Vignone L, et al (1984) Psoriatic arthritis in psoriatic patients. Br J Rheumatol 23: 246–250. 6487929 10.1093/rheumatology/23.4.246

[5] Boehncke WH, Menter A (2013) Burden of disease: psoriasis and psoriatic arthritis. Am J Clin Dermatol 14: 377–388. 23771648 10.1007/s40257-013-0032-x

[6] Cortesi PA, Scalone L, D’Angiolella L, Belisari A, Fusco F, et al (2012) Systematic literature review on economic implications and pharmacoeconomic issues of psoriatic arthritis. Clin Exp Rheumatol 30: S126–131. 23072771

[7] Ghatnekar O, Ljungberg A, Wirestrand LE, Svensson A (2012) Costs and quality of life for psoriatic patients at different degrees of severity in southern Sweden - a cross-sectional study. Eur J Dermatol 22: 238–245. 22361745 10.1684/ejd.2011.1635

[8] Helmick CG, Sacks JJ, Gelfand JM, Bebo B Jr, Lee-Han H, et al (2013) Psoriasis and psoriatic arthritis: a public health agenda. Am J Prev Med 44: 424–426. 23498110 10.1016/j.amepre.2013.01.004 PMC4617774

[9] Kimball AB, Guerin A, Tsaneva M, Yu AP, Wu EQ, et al (2011) Economic burden of comorbidities in patients with psoriasis is substantial. J Eur Acad Dermatol Venereol 25: 157–163. 20561129 10.1111/j.1468-3083.2010.03730.x

[10] Levy AR, Davie AM, Brazier NC, Jivraj F, Albrecht LE, et al (2012) Economic burden of moderate to severe plaque psoriasis in Canada. Int J Dermatol 51: 1432–1440. 23171010 10.1111/j.1365-4632.2011.05359.x

[11] Chandran V, Raychaudhuri SP (2010) Geoepidemiology and environmental factors of psoriasis and psoriatic arthritis. J Autoimmun 34: J314–321. 20034760 10.1016/j.jaut.2009.12.001

[12] Parisi R, Symmons DP, Griffiths CE, Ashcroft DM (2013) Global epidemiology of psoriasis: a systematic review of incidence and prevalence. J Invest Dermatol 133: 377–385. 23014338 10.1038/jid.2012.339

[13] Mease PJ, Gladman DD, Papp KA, Khraishi MM, Thaci D, et al.. (2013) Prevalence of rheumatologist-diagnosed psoriatic arthritis in patients with psoriasis in European/North American dermatology clinics. J Am Acad Dermatol. 10.1016/j.jaad.2013.07.023 23981683

[14] Ogdie A, Langan S, Love T, Haynes K, Shin D, et al (2013) Prevalence and treatment patterns of psoriatic arthritis in the UK. Rheumatology (Oxford) 52: 568–575. 23221331 10.1093/rheumatology/kes324 PMC3573270

[15] Prey S, Paul C, Bronsard V, Puzenat E, Gourraud PA, et al (2010) Assessment of risk of psoriatic arthritis in patients with plaque psoriasis: a systematic review of the literature. J Eur Acad Dermatol Venereol 24 Suppl 2 31–35. 20443998 10.1111/j.1468-3083.2009.03565.x

[16] Asgari MM, Wu JJ, Gelfand JM, Salman C, Curtis JR, et al (2013) Validity of diagnostic codes and prevalence of psoriasis and psoriatic arthritis in a managed care population, 1996–2009. Pharmacoepidemiol Drug Saf 22: 842–849. 23637091 10.1002/pds.3447 PMC3720770

[17] Icen M, Crowson CS, McEvoy MT, Gabriel SE, Maradit Kremers H (2008) Potential misclassification of patients with psoriasis in electronic databases. J Am Acad Dermatol 59: 981–985. 18835060 10.1016/j.jaad.2008.08.034 PMC2840380

[18] Seminara NM, Abuabara K, Shin DB, Langan SM, Kimmel SE, et al (2011) Validity of The Health Improvement Network (THIN) for the study of psoriasis. Br J Dermatol 164: 602–609. 21073449 10.1111/j.1365-2133.2010.10134.x PMC3064479

[19] Love TJ, Cai T, Karlson EW (2011) Validation of psoriatic arthritis diagnoses in electronic medical records using natural language processing. Semin Arthritis Rheum 40: 413–420. 20701955 10.1016/j.semarthrit.2010.05.002 PMC3691811

[20] Singh JA, Holmgren AR, Krug H, Noorbaloochi S (2007) Accuracy of the diagnoses of spondylarthritides in veterans affairs medical center databases. Arthritis Rheum 57: 648–655. 17471541 10.1002/art.22682

[21] Englund M, Joud A, Geborek P, Felson DT, Jacobsson LT, et al (2010) Prevalence and incidence of rheumatoid arthritis in southern Sweden 2008 and their relation to prescribed biologics. Rheumatology (Oxford) 49: 1563–1569. 20444855 10.1093/rheumatology/keq127

[22] Haglund E, Bremander AB, Petersson IF, Strombeck B, Bergman S, et al.. (2011) Prevalence of spondyloarthritis and its subtypes in southern Sweden. Ann Rheum Dis. 10.1136/ard.2010.141598 21288961

[23] Joud A, Petersson IF, Englund M (2012) Low back pain: epidemiology of consultations. Arthritis Care Res (Hoboken) 64: 1084–1088. 22337573 10.1002/acr.21642

[24] Strombeck B, Englund M, Bremander A, Jacobsson LT, Kedza L, et al.. (2010) Cost of Illness from the Public Payers’ Perspective in Patients with Ankylosing Spondylitis in Rheumatological Care. J Rheumatol. 10.3899/jrheum.100099 20716657

[25] Swedish Health Account (2010). Statistics Sweden. Available: http://www.scb.se/en_/Finding-statistics/Statistics-by-subject-area/National-Accounts/National-Accounts/System-of-Health-Accounts-SHA/. Accessed 20 Dec 2013.

[26] Taylor W, Gladman D, Helliwell P, Marchesoni A, Mease P, et al (2006) Classification criteria for psoriatic arthritis: development of new criteria from a large international study. Arthritis Rheum 54: 2665–2673. 16871531 10.1002/art.21972

[27] Bhutani T, Wong JW, Bebo BF, Armstrong AW (2013) Access to health care in patients with psoriasis and psoriatic arthritis: data from National Psoriasis Foundation survey panels. JAMA Dermatol 149: 717–721. 23783152 10.1001/jamadermatol.2013.133

[28] Olivieri I, D’Angelo S, Padula A, Palazzi C (2008) The challenge of early diagnosis of psoriatic arthritis. J Rheumatol 35: 3–5. 18176985

[29] Hellgren L (1967) Psoriasis: The Prevalence in Sex, Age and Occupational Groups in Sweden. Morphology, Inheritance and Association with other Skin and Rheumatic Diseases. Stockholm: Almqvist & Wiksell.

[30] Gelfand JM, Weinstein R, Porter SB, Neimann AL, Berlin JA, et al (2005) Prevalence and treatment of psoriasis in the United Kingdom: a population-based study. Arch Dermatol 141: 1537–1541. 16365254 10.1001/archderm.141.12.1537

[31] Gelfand JM, Gladman DD, Mease PJ, Smith N, Margolis DJ, et al (2005) Epidemiology of psoriatic arthritis in the population of the United States. J Am Acad Dermatol 53: 573. 16198775 10.1016/j.jaad.2005.03.046

[32] Nossent JC, Gran JT (2009) Epidemiological and clinical characteristics of psoriatic arthritis in northern Norway. Scand J Rheumatol 38: 251–255. 19247847 10.1080/03009740802609558

[33] Pedersen OB, Svendsen AJ, Ejstrup L, Skytthe A, Junker P (2008) The occurrence of psoriatic arthritis in Denmark. Ann Rheum Dis 67: 1422–1426. 18208866 10.1136/ard.2007.082172

[34] Shbeeb M, Uramoto KM, Gibson LE, O’Fallon WM, Gabriel SE (2000) The epidemiology of psoriatic arthritis in Olmsted County, Minnesota, USA, 1982–1991. J Rheumatol 27: 1247–1250. 10813295

[35] Wilson FC, Icen M, Crowson CS, McEvoy MT, Gabriel SE, et al (2009) Time trends in epidemiology and characteristics of psoriatic arthritis over 3 decades: a population-based study. J Rheumatol 36: 361–367. 19208565 10.3899/jrheum.080691 PMC2717703

[36] Ibrahim G, Waxman R, Helliwell PS (2009) The prevalence of psoriatic arthritis in people with psoriasis. Arthritis Rheum 61: 1373–1378. 19790120 10.1002/art.24608

[37] Radtke MA, Reich K, Blome C, Rustenbach S, Augustin M (2009) Prevalence and clinical features of psoriatic arthritis and joint complaints in 2009 patients with psoriasis: results of a German national survey. J Eur Acad Dermatol Venereol 23: 683–691. 19309433 10.1111/j.1468-3083.2009.03159.x

[38] Zachariae H, Zachariae R, Blomqvist K, Davidsson S, Molin L, et al (2002) Quality of life and prevalence of arthritis reported by 5,795 members of the Nordic Psoriasis Associations. Data from the Nordic Quality of Life Study. Acta Derm Venereol 82: 108–113. 12125937 10.1080/00015550252948130

[39] Salvarani C, Lo Scocco G, Macchioni P, Cremonesi T, Rossi F, et al (1995) Prevalence of psoriatic arthritis in Italian psoriatic patients. J Rheumatol 22: 1499–1503. 7473473

